# Explainable Feature-Group-Aware Cross-Attentive Expert Fusion for IoMT Intrusion Detection

**DOI:** 10.3390/s26134293

**Published:** 2026-07-06

**Authors:** Asmatullah Khan, Yang Li, Ijaz Khan, Mian Muhammad Kamal

**Affiliations:** 1 School of Information and Communication Engineering, Changchun University of Science and Technology, Changchun 130022, China; 2019300124@mails.cust.edu.cn; 2 State Key Laboratory of Radio Frequency Heterogeneous Integration, Shenzhen University, Shenzhen 518055, China; khanijaz@szpu.edu.cn; 3 School of Electronic and Communication Engineering, Quanzhou University of Information Engineering, Quanzhou 362000, China; mianmuhammadkamal@qzuie.edu.cn

**Keywords:** Internet of Medical Things (IoMT), cyberattack detection, intrusion detection system, cross-attention fusion, Temporal Convolutional Network, explainable artificial intelligence, FT-Transformer, Mixture of Experts, LIME, SHAP

## Abstract

The Internet of Medical Things (IoMT) has become a core component of the modern healthcare system, but its increasing connectivity also exposes medical networks to diverse cyber threats. Although recent threat detection frameworks have demonstrated strong predictive performance, many still operate as black-box models. They offer limited or no interpretability of their decisions. This paper proposes an explainable hybrid IDS framework for multiclass IoMT intrusion detection. The proposed framework partitions network traffic features into semantically related groups and employs specialized expert networks to learn complementary traffic representations. A gate-balanced Mixture-of-Experts (MoE) routing mechanism adaptively aggregates expert outputs, while a cross-expert self-attention module captures contextual dependencies among expert representations. Furthermore, the proposed framework incorporates multi-level interpretability through SHAP, LIME, and expert-routing analysis to explain both feature contributions and internal decision behavior. We evaluate the proposed framework on two recent IoMT benchmarks, namely CICIoMT2024 and IoMT-TrafficData, under 6-class, 19-class, and 9-class multiclass settings, respectively. On CICIoMT2024, the proposed IDS achieves 99.76% accuracy and an MCC of 0.9951 in the 6-class setting, while attaining 99.07% accuracy and an MCC of 0.9892 in the 19-class setting. On IoMT-TrafficData, the proposed framework achieves 99.92% accuracy and an MCC of 0.9988 in the 9-class setting. The explainability results further show that the model identifies meaningful traffic features and exhibits class-dependent expert specialization, thereby improving transparency in its decisions. These findings confirm that the proposed framework provides an effective and interpretable solution for securing IoMT systems.

## 1. Introduction

The IoMT has fundamentally transformed the modern healthcare system by enabling real-time patient monitoring, smart diagnostics, telemedicine, and faster clinical decision-making. However, the continuous connectivity that greatly enhances healthcare quality also expands the cyberattack surface of the medical system [1,2]. IoMT networks usually integrate a wide range of devices, wireless communication links, protocol diversity, resource-constrained endpoints, and highly sensitive health data, making them attractive targets for various cyber attacks such as denial-of-service, spoofing, reconnaissance, and protocol-specific attacks. As a result, IDSs have become a core and fundamental security mechanism for preserving confidentiality, integrity, availability, and operational continuity in IoMT infrastructure [3,4].

The security vulnerabilities in IoMT environments are particularly critical as its directly related to patient safety and the reliability of healthcare services. Unlike traditional IT systems, IoMT devices usually operate with limited computational power and memory resources, heterogeneous communication protocols, and long-term operational devices make regular firmware or cryptographic updates difficult [5]. These limitations prevent the direct deployment of traditional security measures and create fertile ground for different kinds of cyber attacks, including Man-in-the-Middle (MitM) Distributed Denial-of-Service (DDoS) and other cyber attacks. The impact of successful intrusions extends beyond data breaches, and consequences compromised medical system and devices are a direct threat to human life. This makes IoMT security more critical as compared to conventional network security [6,7,8].

In this context, IDS plays as a critical security layer by monitoring network activity and identifying malicious traffic in real time [8,9]. An effective IDS for IoMT environments must be capable of handling various challenges simultaneously. These challenges include high-dimensional, heterogeneous network feature spaces, diverse and evolving attack types, severe class imbalance between attack and normal traffic, low-latency operational requirements, and the need for interpretability to support clinical and regulatory decision making [10]. Fulfilling these requirements at the same time represents one of the most demanding open problems in cybersecurity research.

Earlier studies have explored IoMT intrusion detection using both conventional ML and early DL methods. Traditional classifiers such as Decision Trees (DTs), Random Forests (RFs), k-Nearest Neighbors (KNN), and Support Vector Machines (SVMs) have shown promising performance on benchmark datasets [10]. However, these methods generally depend on manual feature engineering and often struggle to generalize across heterogeneous protocols and dynamic traffic conditions. Their robustness and performance also tend to decline under concept drift, which is very common in real-world IoMT environments. DL methods have further enhanced feature learning and detection performance. Convolutional Neural Networks (CNNs) can capture local traffic patterns effectively [11], but they are not well-suited for capturing long-term temporal patterns. RNNs such as GRU and LSTM can overcome this limitation partially by learning sequential relationships [12]. However, these models often suffer from limited parallel processing efficiency, and their single-branch design may not fully capture both local temporal patterns and global inter-feature relationships in network traffic.

Transformer-based models have also improved sequence representation learning [13]. Transformer Variants such as Tab Transformer [14] and FT-Transformer [15] have demonstrated strong performance on structured data tasks. However, the direct use of transformer-only architectures in intrusion detection may be computationally expensive, while TCN [16,17], although efficient for causal sequence modeling, may fail to capture broader global dependencies as effectively. Therefore, there is a need for an explainable IoMT IDS framework capable of learning specialized representations from heterogeneous traffic characteristics, adaptively selecting the most relevant feature-group experts, and modeling dependencies among expert representations within a unified architecture.

Although many DL-based IDS models achieve high predictive performance, most of them operate as black-box [18,19]. This limited interpretability and transparency are a major concern in healthcare and critical infrastructure, where explanations are needed for trust, auditing, and regulatory compliance. In real IoMT deployments, security analysts and engineers must understand not only whether an alert is generated, but also which traffic features and learned components contribute to the final decision. These limitations motivate the design of an explainable IoMT intrusion detection framework capable of learning specialized representations from heterogeneous traffic features, adaptively routing traffic instances to relevant experts, modeling inter-expert dependencies, and providing transparent decision-making through multi-level interpretability. In this context, two recent IoMT benchmark CICIoMT2024 and IoMTTraffic-Data are suitable for evaluating such architectures under realistic and diverse IoMT traffic conditions.

### 1.1. Contribution

To overcome the identified gaps and limitations, this paper introduced an explainable hybrid DL IDS framework for IoMT. The key contributions of this study are summarized as follows:

1. We propose an explainable intrusion detection framework for IoMT environments that combines feature-group expert learning, adaptive Mixture-of-Experts (MoE) routing, and cross-expert self-attention to capture diverse traffic characteristics and improve multiclass intrusion detection performance.
2. We introduce a feature-group-aware learning strategy that automatically partitions network traffic features into semantically related groups and employs specialized expert networks to learn complementary representations from temporal, protocol, size, statistical, and other traffic attributes.
3. We develop a gate-balanced MoE routing mechanism that dynamically assigns expert importance through adaptive routing probabilities while incorporating balance and entropy regularization to encourage effective expert utilization and prevent expert collapse during training.
4. We incorporate a cross-expert self-attention module that models contextual dependencies among expert representations, enabling the framework to capture complementary relationships across multiple feature groups and generate enriched traffic embeddings for intrusion detection.
5. We enhance the explainability of the proposed framework by integrating SHAP and LIME to provide complementary global feature importance analysis and local instance-level explanations, while expert-routing analysis offers additional insight into the decision-making process of the MoE classifier, thereby improving the transparency and trustworthiness of intrusion detection decisions.

### 1.2. Organization

The remaining sections of this paper is organized as follows: Section 2 discusses the related work. Section 3 describes the proposed IDS framework and training procedure. Section 4 presents the implementation setup, datasets, preprocessing steps, and evaluation metrics. Section 5 presents the performance outcomes and discusses the key findings. Finally, Section 6 concludes the paper and suggests future research directions.

## 2. Related Work

In recent years, many ML and DL approaches have been proposed to improve intrusion detection in IoMT environments using a variety of benchmark datasets. In ref. [20], the authors introduced an IoMT IDS framework that combine convolutional and recurrent neural network components together with reinforcement learning (RL), namely Q-Network (DQN) and Proximal Policy Optimization (PPO), to detect evolving cyber threats. They used Enhanced Mutual Information Feature Selection on the CICIoMT2024, their hybrid model learned both temporal and spatial traffic patterns, achieved 99.58% accuracy in binary classification task, and 77.73% accuracy in 18-class multiclass detection. Similarly, ref. [21] presented a hybrid IoMT security framework integrating CNN, LSTM, and RL, reporting 99.49% accuracy in binary, 99.12% in 6-class, and 98.56% in 19-class classification. A simpler LSTM-based architecture was proposed in [22] for attack classification on the CICIoMT2024 dataset, where it achieved 98% accuracy in 19-class classification. Beyond IoMT-specific studies, ref. [23] proposed a hybrid CNN-LSTM framework for general IoT threat detection and evaluated it on the IoT-23, CICIDS2017, and N-BaIoT datasets, obtaining 95% accuracy on IoT-23 and 99% accuracy on both CICIDS2017 and N-BaIoT.

Transformer-based methods have also gained increasing attention. In ref. [24], the authors proposed the Memory Feedback Transformer (MF-Transformer), which combines MF-LSTM into all Transformer layers to better learn both spatial and temporal dependencies. The model was evaluated on the ECU-IoHT, WUSTL-EHMS-2020, and X-IIoTID datasets, achieving 99.88%, 99.42%, and 99.12% accuracy for signature detection, and 99.98%, 99.71%, and 99.18% for anomaly detection, respectively. Likewise, ref. [25] introduced a lightweight Transformer-based IDS for medical IoT that combines flow and packet-level features through depth-wise separable convolutions and a two-layer Transformer encoder. Using the IoT Healthcare and IoMT-TrafficData datasets, the model achieved up to 97.9% F1-score in multiclass classification. In ref. [26], a Transformer-based framework was proposed specifically for spoofing attack detection in IoMT, achieving 99.71% accuracy in binary classification. In ref. [27], the authors combined TabTransformer and Random Forest to detect attacks such as DDoS and DoS in biometric healthcare systems using the CICIoMT2024 dataset, and reported 99.5% classification accuracy.

More recently, Mixture-of-Experts (MoE) and hybrid expert-based methods have also been explored. MedMixtral 8x7B, proposed in [28], is a medical large language model based on an MoE architecture for IoMT-enabled e-healthcare. Although it is not primarily an intrusion detection model, it demonstrates the potential of expert-based architectures in resource-constrained IoMT settings through an offloading strategy that improves deployment feasibility and user privacy. In ref. [29], an IoT threat detection framework combining ConvNeXt, sparse BiLSTM experts, and a symmetric linear routing-based sparse MoE mechanism.They utilized CIC-IDS2018, BoT-IoT, and TON-IoT, achieving 94.08%, 99.99%, and 99.78% accuracy, respectively. Another related study [30] proposed a CPS-oriented intrusion detection framework that integrates signature-based intrusion detection with ML and DL techniques, including an MoE model and a context-aware CPS-SNORT ruleset for deep packet inspection of G-code instructions. The reported results exceeded 99% accuracy for known attack detection, 85% accuracy in semi-supervised settings, and 99.9% accuracy for behavioral anomaly detection using LSTM. Finally, ref. [31] addressed IoMT ransomware detection using a hybrid framework that combines Random Forest or XGBoost with Temporal Convolutional Networks (TCNs). Experiments on a synthetic IoMT ransomware dataset showed that both XGBoost+TCN and Random Forest+TCN outperformed their standalone baselines, with the best result reaching 91.24% accuracy. Table 1 summarizes recent neural network approaches for securing IoMT systems.

## 3. Proposed Architecture

This section presents the proposed framework for explainable IoMT intrusion detection. The framework is designed to improve intrusion detection performance while maintaining transparency in its decision-making process. The individual components of the framework are described in detail in the following subsections.

### 3.1. Overview of the Proposed Framework

Let the preprocessed traffic sample be represented by $x\in R^{d}$, where *d* denotes the number of input features. The proposed framework first partitions the input feature vector into multiple semantically related feature groups, which are subsequently processed by dedicated expert networks to learn specialized traffic representations. The resulting expert outputs are aggregated through a gate-balanced MoE routing mechanism that adaptively determines the contribution of each expert for a given traffic instance. To further capture dependencies among expert representations, a cross-expert self-attention module is employed to model inter-expert interactions and complementary feature relationships. The MoE representation and attention-enhanced representation are then fused and passed to a multiclass classification module for final intrusion detection. Furthermore, SHAP, LIME, and expert-routing analysis are incorporated to provide both feature-level and model-level interpretability.

### 3.2. Input Layer

Let the raw traffic sample be represented by

$$x={\left[x_{1},x_{2},\ldots ,x_{d}\right]}^{⊤}\in R^{d}$$

where *d* denotes the number of input features. To improve training stability and mitigate feature skewness [32], selected non-negative heavy-tailed features are transformed using

(1) $$x_{i}^{\prime }=\log\left(1+x_{i}\right)$$

Subsequently, feature standardization is applied as

(2) $${\hat{x}}_{i}=\frac{x_{i}^{\prime }-\mu_{i}}{\sigma_{i}}$$

where $\mu_{i}$ and $\sigma_{i}$ denote the mean and standard deviation estimated from the training data. The same transformation is applied to the validation and test sets to avoid information leakage. The resulting standardized feature vector

$$\hat{x}={\left[{\hat{x}}_{1},{\hat{x}}_{2},\ldots ,{\hat{x}}_{d}\right]}^{⊤}$$

is used as the input to the proposed framework. The normalized feature space facilitates stable optimization and serves as the basis for subsequent feature-group expert learning and adaptive expert routing.

### 3.3. Feature-Group Expert Learning Module

To capture diverse characteristics of IoMT traffic, the proposed framework partitions the normalized feature vector into multiple semantically related feature groups. Specifically, features are automatically assigned to five disjoint groups representing temporal characteristics, protocol information, size and rate measurements, statistical attributes, and miscellaneous features. Let

$$G=\left\{G_{\mathrm{time}},G_{\mathrm{proto}},G_{\mathrm{size}},G_{\mathrm{stats}},G_{\mathrm{other}}\right\}$$

denote the resulting set of feature groups. For each group $G_{k}$, a dedicated expert network is employed to learn specialized representations from the corresponding subset of features,

$$x_{k}={\hat{x}}_{i}|i\in G_{k},$$

where $x_{k}$ denotes the input to the *k*-th expert. This feature-group-aware design promotes expert specialization and enables the framework to learn complementary representations from different aspects of IoMT network traffic [33,34].

### 3.4. Gate-Balanced Mixture-of-Experts Routing

For each feature group $x_{k}$, the proposed framework employs a dedicated expert network to learn a specialized representation. Each expert consists of two fully connected layers with Layer Normalization and Dropout regularization. The output of the (k)-th expert is given by

(3) $$h_{k}=E_{k}\left(x_{k}\right),$$

where $E_{k}\left(\cdot \right)$ denotes the expert network associated with feature group $G_{k}$, and $h_{k}\in R^{m}$ represents the learned expert representation [34]. The outputs of all experts are subsequently stacked to form the expert representation matrix

$$H=[h_{1},h_{2},\ldots ,h_{K}]$$

where *K* denotes the total number of experts. Rather than assigning equal importance to all experts, the proposed framework employs a gating network that dynamically determines the contribution of each expert for a given traffic instance.

#### 3.4.1. Gating Network

To adaptively determine the contribution of each expert, the proposed framework employs a gating network that generates expert routing probabilities from the input feature vector. The routing weights are computed as

(4) $$g=\mathrm{softmax}\left(\frac{W_{g}\hat{x}+b_{g}}{\tau }\right),$$

where $W_{g}$ and $b_{g}$ are trainable parameters, τ is a temperature coefficient, and $g=[g_{1},g_{2},\ldots ,g_{K}]$ denotes the routing probabilities assigned to the *K* experts. The Softmax operation ensures that the expert weights sum to one, enabling adaptive expert selection for each traffic sample.

#### 3.4.2. Gate Balance Regularization

To prevent the gating network from consistently selecting a small subset of experts, the proposed framework incorporates a gate balance regularization mechanism. Let g denote the routing probabilities obtained from Equation (4). The average routing probability across a mini-batch is computed as

(5) $$\bar{g}=\frac{1}{N}\sum_{i=1}^{N}g_{i},$$

where *N* denotes the batch size. A balance loss is introduced to encourage uniform expert utilization,

(6) $$L_{\mathrm{bal}}=\sum_{k=1}^{K}{\left({\bar{g}}_{k}-\frac{1}{K}\right)}^{2},$$

where *K* is the number of experts. In addition, an entropy-based regularization term is employed to discourage overly confident routing decisions,

(7) $$L_{\mathrm{ent}}=1+\frac{1}{N\log K}\sum_{i=1}^{N}\sum_{k=1}^{K}g_{ik}\log\left(g_{ik}\right),$$

The final gate regularization objective is given by

(8) $$L_{\mathrm{reg}}=\lambda_{b}L_{\mathrm{bal}}+\lambda_{e}L_{\mathrm{ent}},$$

where $\lambda_{b}$ and $\lambda_{e}$ control the contributions of the balance and entropy regularization terms, respectively [34,35].

#### 3.4.3. Expert Mixture Aggregation

The final expert representation is obtained by combining the outputs of all experts according to the routing probabilities generated by the gating network [36]. Specifically, the MoE representation is computed as

(9) $$h_{\mathrm{MoE}}=\sum_{k=1}^{K}g_{k}h_{k},$$

where $g_{k}$ denotes the routing probability assigned to the *k*-th expert and $h_{k}$ represents the corresponding expert output. Through this adaptive weighted aggregation, the framework dynamically emphasizes the most relevant expert representations for each traffic sample while preserving complementary information from multiple feature groups.

### 3.5. Cross-Expert Self-Attention Module

Although the MoE aggregation captures the relative importance of individual experts, dependencies may still exist among expert representations. To model such interactions, the proposed framework incorporates a cross-expert self-attention module that learns contextual relationships among expert outputs. Given the stacked expert representation matrix H, self-attention is computed as

(10) A=Attention(Q,K,V),

where Q, K, and V denote the query, key, and value projections of H, respectively. The resulting attention output is combined with the original expert representations through a residual connection followed by layer normalization,

(11) $$H^{\prime }=\mathrm{LayerNorm}\left(A+H\right),$$

The final attention-enhanced representation is obtained using global average pooling,

(12) $$h_{att}=\mathrm{GAP}\left(H^{\prime }\right),$$

where $h_{att}$ summarizes the contextual information learned across all expert representations [37,38].

### 3.6. Fusion and Multiclass Classification

To jointly exploit the complementary information learned through adaptive expert routing and cross-expert attention, the corresponding representations are concatenated to form a unified feature representation,

(13) $$h_{f}=\left[h_{\mathrm{MoE}};h_{att}\right],$$

where $h_{\mathrm{MoE}}$ and $h_{att}$ denote the MoE and attention-enhanced representations, respectively. The fused representation is subsequently processed by fully connected layers to learn a compact discriminative embedding,

(14) $$z=\phi (W_{f}h_{f}+b_{f}),$$

where ϕ(·) denotes the nonlinear activation function and $W_{f}$ and $b_{f}$ are trainable parameters. Finally, the probability distribution over the intrusion classes is obtained through a Softmax classifier,

(15) $$\hat{y}=\mathrm{Softmax}\left(W_{c}z+b_{c}\right),$$

where $\hat{y}$ represents the predicted class probabilities.

**Figure 1:**
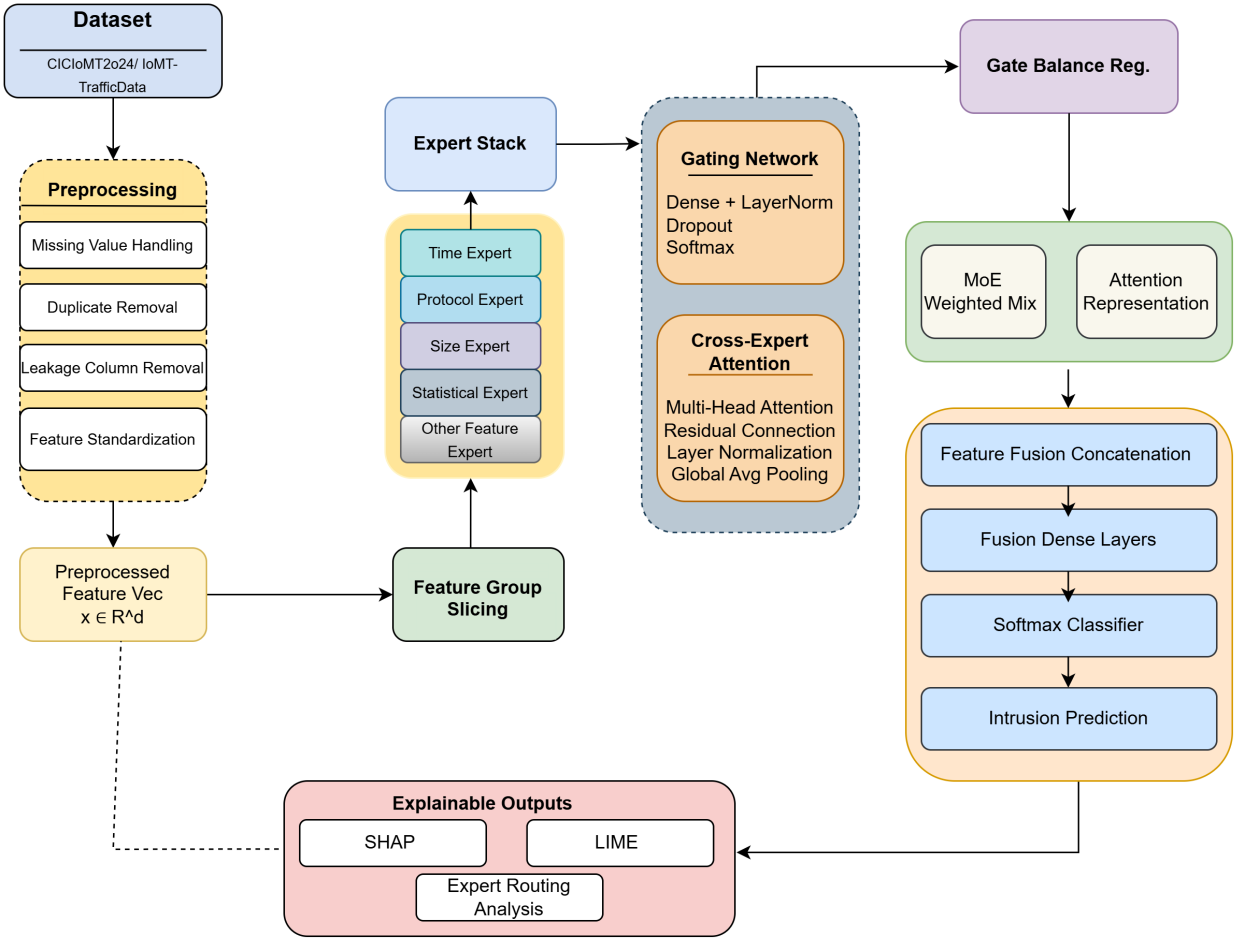
Diagram of the proposed framework.

Figure 1 illustrates the structural diagram, while Algorithm 1 presents the step-by-step process of the proposed framework.
**Algorithm 1** HybridMoE-IDS for IoMT Intrusion Detection**Require:** Preprocessed IoMT traffic dataset $D={\left\{\left({\hat{x}}_{i},y_{i}\right)\right\}}_{i=1}^{N}$, feature groups $G=\{G_{1},\ldots ,G_{K}\}$, number of experts *K*, trainable parameters θ**Ensure:** Trained HybridMoE-IDS model  1:**for** each epoch **do**  2:    **for** each mini-batch $B=\{\left(\hat{X},Y\right)\}\subset D$ **do**  3:        **for** k=1 to *K* **do**  4:              Extract group-specific features: $X_{k}\leftarrow \hat{X}\left[:,G_{k}\right]$  5:              Compute expert representation: $H_{k}\leftarrow E_{k}\left(X_{k}\right)$  6:        **end for**  7:        Stack expert outputs: $H\leftarrow [H_{1},H_{2},\ldots ,H_{K}]$  8:        Compute routing probabilities: $G\leftarrow \mathrm{softmax}(\left(W_{g}\hat{X}+b_{g}\right)/\tau )$  9:        Aggregate expert outputs: $H_{\mathrm{MoE}}\leftarrow \sum_{k=1}^{K}G_{k}H_{k}$10:        Model cross-expert interactions: $H^{\prime }\leftarrow \mathrm{LayerNorm}\left(\mathrm{MHA}\left(H,H,H\right)+H\right)$11:        Pool attention output: $H_{att}\leftarrow \mathrm{GAP}\left(H^{\prime }\right)$12:        Fuse representations: $Z_{f}\leftarrow \left[H_{\mathrm{MoE}};H_{att}\right]$13:        Predict class probabilities: $\hat{Y}\leftarrow \mathrm{softmax}\left(W_{c}Z_{f}+b_{c}\right)$14:        Incorporate gate-balance regularization $L_{\mathrm{reg}}$ during optimization15:        Update θ using backpropagation and Adam optimizer16:    **end for**17:**end for**18:Generate explainability outputs using SHAP, LIME, and expert-routing analysis

### 3.7. Interpretability of the Proposed Framework

To improve transparency, the proposed framework is analyzed using a combination of post hoc interpretability techniques and internal model behavior analysis. It is important to note that SHAP and LIME are employed strictly as post hoc explanation tools after model training and are not involved in feature selection, model optimization, or any part of the learning process. Specifically, SHAP is used to quantify global and local feature attributions, while LIME provides instance-level explanations for representative predictions. In addition, the gating outputs of the Mixture-of-Experts (MoE) module are examined to analyze expert utilization patterns across different attack categories. This combined analysis enables a comprehensive interpretation of the model at the feature level, sample level, and expert-routing level, providing insights into the decision behavior of the proposed intrusion detection framework.

#### 3.7.1. SHAP-Based Feature Attribution

SHAP explains model predictions by assigning each input feature a contribution value derived from Shapley values in cooperative game theory. For a given sample, the SHAP value of feature *i* measures how much that feature contributes to the deviation of the prediction from the baseline output. Formally, the contribution of feature *i* can be written as

(16) $$\phi_{i}\left(f,x\right)=\sum_{S\subseteq F\setminus \{i\}}\frac{|S|!(M-|S|-1)!}{M!}\left[f_{S\cup \{i\}}\left(x\right)-f_{S}\left(x\right)\right]$$

where *F* denotes the full feature set, M=|F|, and *S* is a subset of features excluding *i*. In the proposed framework, SHAP is used to identify the most influential traffic attributes for attack discrimination, generate global feature-importance rankings, and analyze class-specific decision behavior. This is particularly useful in IoMT IDS settings, where understanding dominant traffic indicators is essential for model trust and security analysis [39,40,41].

#### 3.7.2. LIME-Based Local Explanation

LIME explains an individual prediction by approximating the complex model locally with an interpretable surrogate model. Instead of interpreting the full nonlinear decision surface, LIME focuses on the neighborhood around a target sample and highlights the features that most strongly support or oppose the predicted class. The local explanation is obtained by solving

(17) $$\xi \left(x\right)=\arg\min_{g\in G}L\left(f,g,\pi_{x}\right)+\omega \left(g\right),$$

where *f* represents the original model, *g* denotes an interpretable surrogate selected from the model family *G*, $\pi_{x}$ defines the local neighborhood around sample x, L measures the approximation error, and ω(g) controls model complexity. In this work, LIME is used to provide case-specific explanations for representative correctly classified and misclassified samples, thereby showing which traffic features drive individual IDS decisions. Such local explanations complement SHAP by offering human-readable evidence for specific alerts [40,41,42].

#### 3.7.3. Expert Routing and Gating Output Analysis

In addition to feature-level explanations, the proposed framework provides model-level interpretability through the analysis of expert routing behavior. Specifically, the gating network produces a routing probability vector for each traffic sample, indicating the relative contribution of individual experts to the final prediction. By examining the distribution of routing probabilities across different intrusion classes, it is possible to identify expert specialization patterns and understand how the framework allocates attention to different feature groups. For a given traffic sample, the routing probability assigned to the *k*-th expert is represented by $g_{k}$, where larger values indicate greater reliance on the corresponding expert. The average routing probability for each expert can be computed across samples belonging to the same attack category, enabling the visualization of expert utilization patterns. Such analysis provides insights into which feature groups are most influential for detecting specific intrusion types.

Unlike conventional black-box classifiers, the proposed framework exposes its internal decision process through the gating mechanism. Consequently, expert routing analysis complements SHAP and LIME explanations by revealing not only which features contribute to a prediction but also which expert representations are primarily responsible for the final decision. This additional level of transparency facilitates model auditing and improves the interpretability of intrusion detection outcomes in IoMT environments [33,43].

## 4. Experimental Design

This section presents the benchmark datasets, preprocessing strategy, implementation settings, and evaluation metrics used to assess the proposed IDS framework. All experiments were conducted under multiclass intrusion detection settings in order to evaluate the capability of the model to distinguish benign traffic from multiple attack categories in IoMT environments.

### 4.1. Benchmark Datasets

Two recent IoMT datasets were used in this study, namely CICIoMT2024 and IoMT-TrafficData. CICIoMT2024 is a recent benchmark dataset developed for security assessment in IoMT environments. It was generated from an IoMT testbed containing real and simulated healthcare-related devices and includes multiple cyberattack scenarios collected over different communication settings [5]. The dataset covers diverse attack categories and protocol conditions, which makes it suitable for evaluating intrusion detection models under heterogeneous IoMT traffic behavior. In this work, CICIoMT2024 was used in both 6 classes and 19 classes multiclass settings in order to evaluate the proposed framework at different levels of attack granularity. IoMT-TrafficData is another recent dataset specifically introduced for benchmarking intrusion detection in IoMT networks. It contains benign traffic together with multiple attack classes and provides flow-based traffic representations that are suitable for ML and DL based intrusion detection [44]. In this study, the IP-based flow representation was used because it offers structured flow-level features appropriate for the proposed hybrid architecture.

Table 2 indicates that both CICIoMT2024 and IoMT-TrafficData exhibit substantial class imbalance. For CICIoMT2024, the DDoS and DoS classes account for approximately 66.63% and 25.32% of all samples, respectively, whereas the Spoofing class represents only 0.20% of the dataset, resulting in a largest-to-smallest class ratio exceeding 328:1. Similarly, IoMT-TrafficData is dominated by the camoverflow class (50.57%), while arpspoofing and slowread constitute only 0.35% and 0.28% of the samples, respectively, yielding a largest-to-smallest class ratio of approximately 182:1. Rather than modifying the original data distribution through over or under-sampling techniques, this study preserves the natural class proportions to reflect realistic IoMT network traffic conditions. To ensure a fair assessment under these imbalanced settings, the proposed framework is evaluated using macro-averaged precision, recall, and F1-score, where each class contributes equally to the final metric regardless of its frequency. Consequently, the reported performance reflects the model’s effectiveness across both majority and minority attack categories instead of being dominated by the most frequent classes.

### 4.2. Preprocessing Strategy

A dataset-aware preprocessing pipeline was applied before model training. First, invalid entries such as missing values, NaN values, and infinite values were handled during the data cleaning stage. Duplicate samples were removed where necessary to reduce redundancy and improve data consistency. For CICIoMT2024, the official data split was preserved as the base experimental setting. In the 6-class experiments, fine-grained labels were mapped into broader attack families to form a coarse multiclass benchmark. In the 19-class experiments, labels were harmonized into a consistent multiclass taxonomy in order to support stable evaluation across the provided split. For IoMT-TrafficData, the multiclass target was defined using the traffic label, while the helper binary field is_attack was removed from the input features to avoid target leakage. After target separation, only numerical attributes were retained for model input, resulting in a fixed-length tabular feature representation compatible with the proposed framework. To improve feature stability, a selective log(1+x) transformation was applied to non-negative heavy-tailed attributes. Afterward, all retained numerical features were scaled using Z-score normalization. IoMT-TrafficData was first divided into a training and a test subset. Using a stratified split, the validation set was then created from the training portion only. For CICIoMT2024, validation data were drawn from the training portion while preserving the official train-test split. This preprocessing procedure ensured a leakage-aware and reproducible experimental workflow.

### 4.3. Implementation Details and Hyperparameter Settings

The proposed framework was implemented in a Python 3.12.7 environment using TensorFlow 2.19.0/Keras 3.10.0 for network construction and training, scikit-learn was used for preprocessing and performance evaluation, while we employed SHAP and LIME for interpretability analysis. All experiments were carried out in a Jupyter Notebook 7.2.2 on a machine equipped with 16 GB RAM. The model was trained using the Adam optimizer with a learning rate of $3\times 10^{-4}$ and a batch size of 64. A dropout rate of 0.25 was used for regularization. The maximum number of training epochs was set according to the dataset and experiment type, while model selection was guided by validation performance. Early stopping and learning-rate scheduling were employed when necessary to improve convergence stability and reduce unnecessary training epochs. For the proposed framework, the expert representation dimension, fusion dimension, and routing configuration were selected empirically based on validation performance. In the main experiments, the model employed feature-group-specific expert networks, gate-balanced Mixture-of-Experts (MoE) routing, and a cross-expert self-attention module to capture complementary traffic characteristics. Gate-balancing regularization was incorporated during training to encourage stable expert utilization and prevent expert dominance across different traffic classes. The principal hyperparameters used in this work are summarized in Table 3.

### 4.4. Evaluation Metrics

The proposed framework was evaluated on standard multiclass classification metrics, including accuracy, recall, precision, and F1-score. Because recent IoMT-specific datasets often shows class imbalance, both macro-level and weighted performance measures were considered during analysis.

Accuracy is defined as

(18) $$\mathrm{Accuracy}=\frac{TP+TN}{TP+TN+FP+FN},$$

where TP, TN, FP, and FN represent true positives, true negatives, false positives, and false negatives predictions respectively.

Precision and recall are computed as

(19) $$\mathrm{Precision}=\frac{TP}{TP+FP},$$

(20) $$\mathrm{Recall}=\frac{TP}{TP+FN}.$$

The F1-score, which provides a balance between precision and recall, is given by

(21) $$F1=\frac{2\times \mathrm{Precision}\times \mathrm{Recall}}{\mathrm{Precision}+\mathrm{Recall}}.$$

## 5. Results and Discussion

This section presents the experimental results of the proposed IDS framework on the benchmark IoMT datasets and discusses the corresponding interpretability outcomes. The analysis is organized to first examine the optimization behavior of the model, followed by predictive performance evaluation, and then multi-level explainability analysis. In addition to standard classification results, feature-level and expert-level interpretation are also provided to better understand how the proposed framework reaches its decisions.

### 5.1. 6-Class Results on CICIoMT2024

#### 5.1.1. Classification Performance Analysis

**Figure 2:**
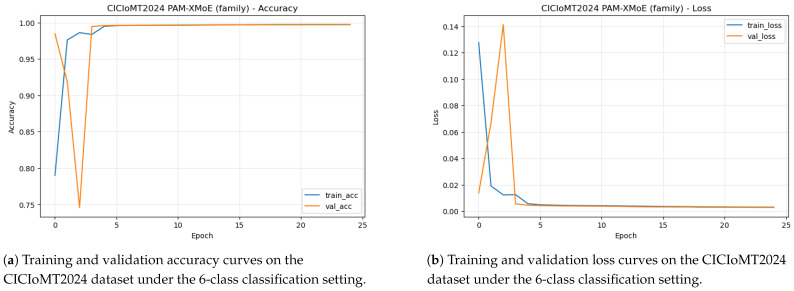
Training behavior of the proposed framework on the CICIoMT2024 dataset under the 6-class classification setting.

**Figure 3:**
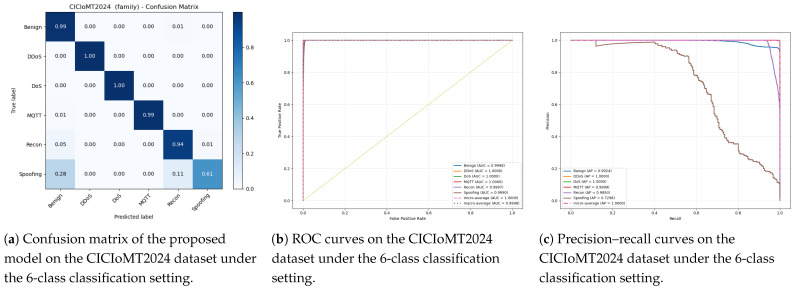
Performance evaluation of the proposed model on the CICIoMT2024 6-class classification task. (**a**) Confusion matrix. (**b**) ROC curves. (**c**) Precision–recall curves.

This subsection provides the 6-class evaluation of the proposed IDS on the CICIoMT2024 dataset. In this setting, the model is required to distinguish benign traffic from six broad traffic families, namely *Benign*, *DDoS*, *DoS*, *MQTT*, *Recon*, and *Spoofing*. Compared with the fine-grained (19-class) setting, 6-class classification provides a more general view of the ability of the model to separate major attack categories while still preserving meaningful multiclass discrimination. Figure 2a illustrates the training and validation accuracy curves, while Figure 2b shows the corresponding loss curves. The proposed model converges rapidly within the first few epochs, with both training and validation accuracy increasing toward near-saturated values and both loss curves decreasing sharply. Although a brief fluctuation appears in the early validation trajectory, the model quickly stabilizes and maintains highly consistent optimization behavior afterward. This pattern indicates that the proposed hybrid architecture learns discriminative 6-class IoMT traffic representations efficiently and does not exhibit sustained signs of overfitting. The close agreement between the training and validation curves further suggests that the learned representation generalizes well across the validation subset. The normalized confusion matrix in Figure 3a shows that the proposed framework achieves very strong discrimination for most traffic families. In particular, *DDoS* and *DoS* are classified almost perfectly, while *Benign*, *MQTT*, and *Recon* also maintain high recall. The main residual difficulty is concentrated in the *Spoofing* family, which is more frequently confused with *Benign* and, to a lesser extent, *Recon*. This indicates that spoofing traffic shares greater statistical overlap with normal and reconnaissance-related behavior than the more dominant attack families. Overall, the 6-class setting confirms that the proposed model is highly effective at coarse-grained intrusion discrimination, with the main limitation restricted to the minority spoofing class. Furthermore, the ROC and one-vs-rest Precision–Recall curves in Figure 3b,c further support this conclusion. The ROC results are near-saturated for all classes, with a macro-average AUC of approximately 0.9998 and a micro-average AUC of 1.0000, indicating extremely strong class separability. The Precision–Recall curves provide a more informative view under class imbalance and show that most classes retain very high average precision, whereas *Spoofing* exhibits a noticeably weaker profile. This is consistent with the confusion matrix and confirms that the remaining 6-class challenge lies in separating spoofing behavior from benign and reconnaissance traffic rather than in distinguishing the dominant attack families. Table 4 compares the proposed IDS with Logistic Regression (LR), AdaBoost (AB), and Deep Neural Network (DNN) on the CICIoMT2024 dataset [5] under both 6-class and 19-class settings. The proposed model achieves the best performance in all reported metrics, reaching 99.7590% accuracy and 0.9291 F1-score at the 6-class, and 99.0667% accuracy and 0.8429 F1-score at the 19-class setting. These results indicate that the proposed framework provides more reliable and robust multiclass intrusion detection than the baseline models, particularly in the more challenging 19-class classification setting.

Table 5 presents the per-class performance of proposed IDS on the CICIoMT2024 dataset under the 6-class setting. The proposed framework achieves an overall accuracy of 99.76% and an MCC of 0.9951, demonstrating excellent detection capability across the major attack families. The model attains near-perfect precision, recall, and F1-scores for the DDoS, DoS, and MQTT classes, indicating highly reliable classification of these attack categories. Similarly, strong performance is observed for the Recon class, with an F1-score of 0.9600. Although the Spoofing class exhibits comparatively lower performance, it remains the most challenging category due to its similarity to benign traffic patterns. Overall, the high macro-averaged precision (0.9381), recall (0.9215), and F1-score (0.9291) confirm the effectiveness and robustness of framework for 6-class IoMT intrusion detection.

#### 5.1.2. Explainability and Interpretability Analysis

**Figure 4:**
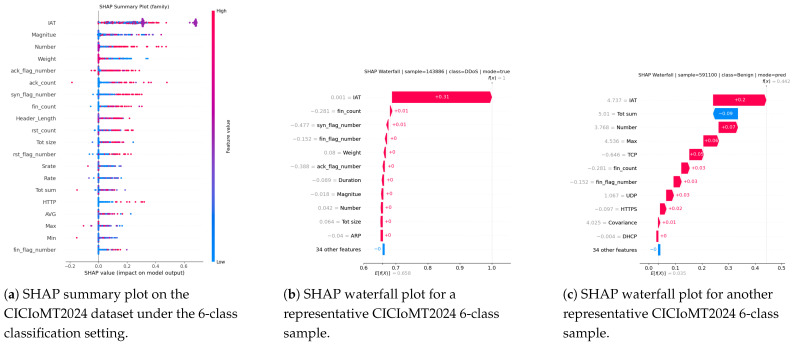
SHAP-based global and local explanations for the CICIoMT2024 dataset under the 6-class classification setting. (**a**) SHAP summary plot showing global feature importance. (**b**,**c**) SHAP waterfall plots illustrating local feature contributions for representative predictions.

**Figure 5:**
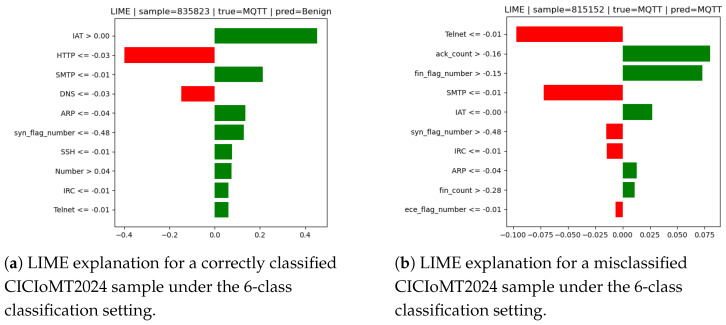
LIME-based local explanations for representative correct and incorrect predictions on the CICIoMT2024 dataset under the 6-class classification setting.

**Figure 6:**
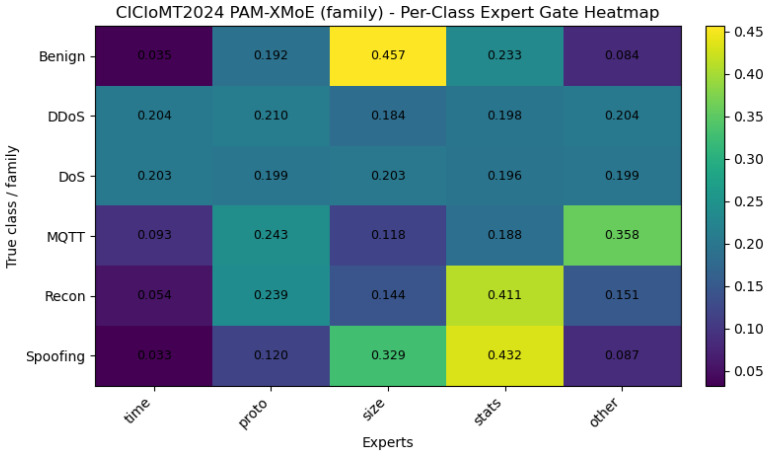
Gate heatmap illustrating expert routing behavior in the CICIoMT2024 under 6-class classification setting.

**Figure 7:**
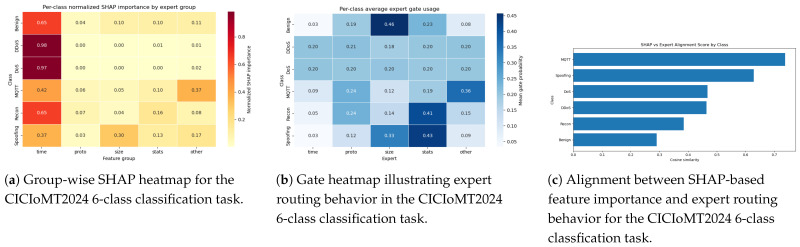
Interpretability analysis of the proposed model on the CICIoMT2024 6-class classification task.

The SHAP summary plot in Figure 4a shows that 6-class classification decisions are driven by a compact group of informative features, with *IAT* emerging as the dominant attribute, followed by variables such as *Magnitude*, *Number*, *Weight*, and several flag- and count-related features. The representative SHAP waterfall plots in Figure 4b,c further demonstrate how these features accumulate to support individual predictions. In the *DDoS* example, *IAT* provides the strongest positive contribution, supported by packet-count and flag-related features, whereas in the benign prediction case, the decision is mainly driven by *IAT*, *Number*, and *Max*, with opposing influence from *Tot sum*. The LIME explanations in Figure 5a,b provide complementary local evidence by showing how timing and protocol-related variables support correct *MQTT* detection in one case and contribute to a benign-side decision in a more difficult sample. Figure 6 shows clear class-dependent routing in the MoE module. *Benign* traffic is routed mainly to the *size* expert, while *MQTT* is associated more with the *other* expert. *Recon* and *Spoofing* rely more on the *stats* expert, whereas *DDoS* and *DoS* exhibit more distributed routing across experts. This suggests that the gating mechanism adapts expert selection according to family-specific traffic characteristics. Figure 7a–c show the relationship between feature-group importance and expert routing across traffic families. The SHAP-group heatmap indicates that the *time* group is dominant for most classes, especially *DDoS* and *DoS*, while *MQTT* and *Spoofing* rely on a more distributed set of groups. The gate heatmap shows complementary class-dependent routing, with *Benign* favoring the *size* expert, *MQTT* the *other* expert, and *Recon* and *Spoofing* the *stats* expert. Figure 7c quantifies the agreement between these views. The highest alignment is observed for *MQTT* and *Spoofing*, while *Benign* and *Recon* show lower alignment. Overall, the results confirm meaningful class-dependent routing and broad consistency between SHAP-based feature importance and internal expert usage.

### 5.2. 19-Class Results on CICIoMT2024

#### 5.2.1. Classification Performance Analysis

**Figure 8:**
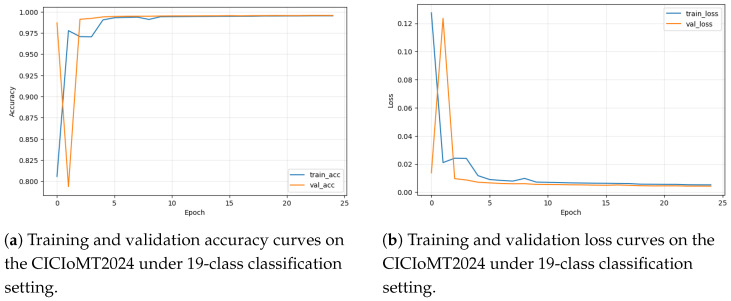
Training behavior of the proposed model on the CICIoMT2024 under a 19-class classification setting.

**Figure 9:**
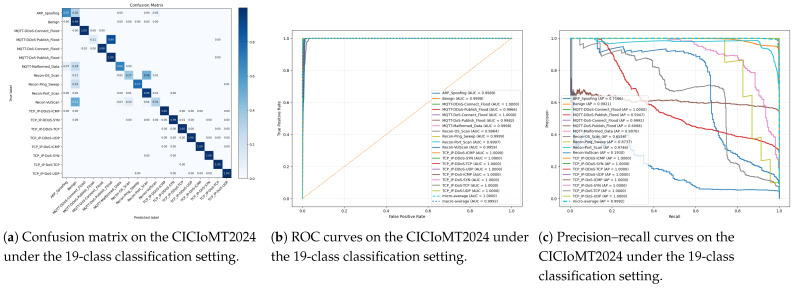
Performance evaluation of the proposed model on the CICIoMT2024 19-class classification task. (**a**) Confusion matrix. (**b**) ROC curves. (**c**) Precision–recall curves.

This subsection evaluates the proposed IDS framework under the harmonized 19-class setting of CICIoMT2024. Compared with the 6-class classification task, 19-class classification is more challenging because the model must distinguish individual attack subtypes with partially overlapping traffic behavior. As shown in Figure 8a,b, the model converges rapidly after a brief early fluctuation in validation performance and then remains stable across the remaining epochs. This behavior indicates effective optimization and suggests that the hybrid IDS framework is able to learn discriminative subtype-level representations without obvious instability. The normalized confusion matrix in Figure 9a shows that the proposed framework performs extremely well on several TCP/IP DoS and DDoS subclasses, many of which are classified almost perfectly. However, the remaining difficulty is concentrated in minority or semantically similar classes. In particular, *ARP_Spoofing* is often confused with *Benign*, *Recon_OS_Scan* is strongly confused with *Recon_Port_Scan*, and *Recon_VulScan* remains one of the most challenging categories. Some confusion is also observed among related MQTT subtypes, especially for publish-flood variants. These results indicate that the proposed model handles dominant fine-grained attack classes very effectively, while subtype-level ambiguity remains the main source of residual error. Furthermore, the ROC and one-vs-rest Precision–Recall curves in Figure 9b,c provide a more complete picture of class separability. The ROC curves remain near-saturated for most classes, with a macro-average AUC of approximately 0.9992, which confirms strong overall discrimination. In contrast, the Precision–Recall curves reveal the real difficulty of minority and behaviorally close classes. While dominant TCP/IP attack types preserve nearly ideal precision–recall behavior, weaker classes such as *Recon_VulScan*, *Recon_OS_Scan*, *ARP_Spoofing*, and certain MQTT subclasses show noticeably lower average precision. Therefore, the 19-class evaluation confirms that the main challenge is not broad attack-family detection, but the separation of minority and closely related subtypes under multiclass imbalance.

Table 6 presents the per class performance of proposed IDS on the CICIoMT2024 dataset under the 19-class classification setting. The proposed framework achieves an overall accuracy of 99.07%, a macro F1-score of 0.8428, and an MCC of 0.9891, demonstrating strong discrimination among closely related attack categories. Most MQTT and TCP/IP attack classes achieve near-perfect precision, recall, and F1-scores. Lower performance is observed for ARP-Spoofing, Recon-OS Scan, Recon-VulScan, and MQTT-DDoS-Publish Flood, indicating the increased difficulty of distinguishing certain minority and highly similar attack subclasses. Hower, the overall results confirm the effectiveness of proposed IDS for 19-class IoMT intrusion detection.

#### 5.2.2. Explainability and Interpretability Analysis

**Figure 10:**
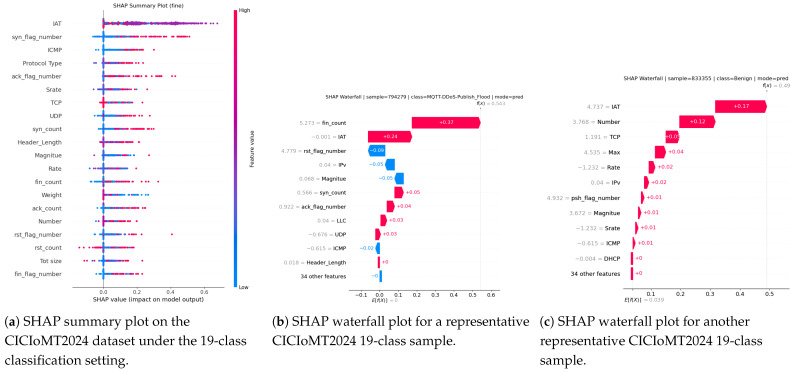
SHAP-based global and local explanations for the CICIoMT2024 dataset under the 19-class classification setting. (**a**) SHAP summary plot showing global feature importance. (**b**,**c**) SHAP waterfall plots illustrating local feature contributions for representative predictions.

**Figure 11:**
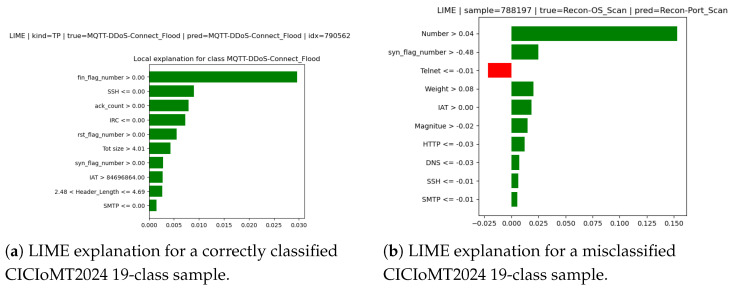
LIME-based local explanations for representative correct and incorrect predictions in the CICIoMT2024 19-class task.

The SHAP summary plot in Figure 10a shows that subtype-level decisions are driven mainly by a compact set of informative traffic attributes, with *IAT* emerging as the dominant feature, followed by variables such as *syn_flag_number*, *ICMP*, *Protocol Type*, and *ack_flag_number*. The representative SHAP waterfall plots in Figure 10b,c further illustrate how these features contribute to individual predictions. In the MQTT-related example, *fin_count* and *IAT* act as the strongest positive contributors, while in the benign prediction case, *IAT*, *Number*, and *TCP*-related features drive the decision. The LIME explanations in Figure 11a,b provide additional local support for these observations by showing which features support or oppose the final class assignment in representative correct and difficult samples.

**Figure 12:**
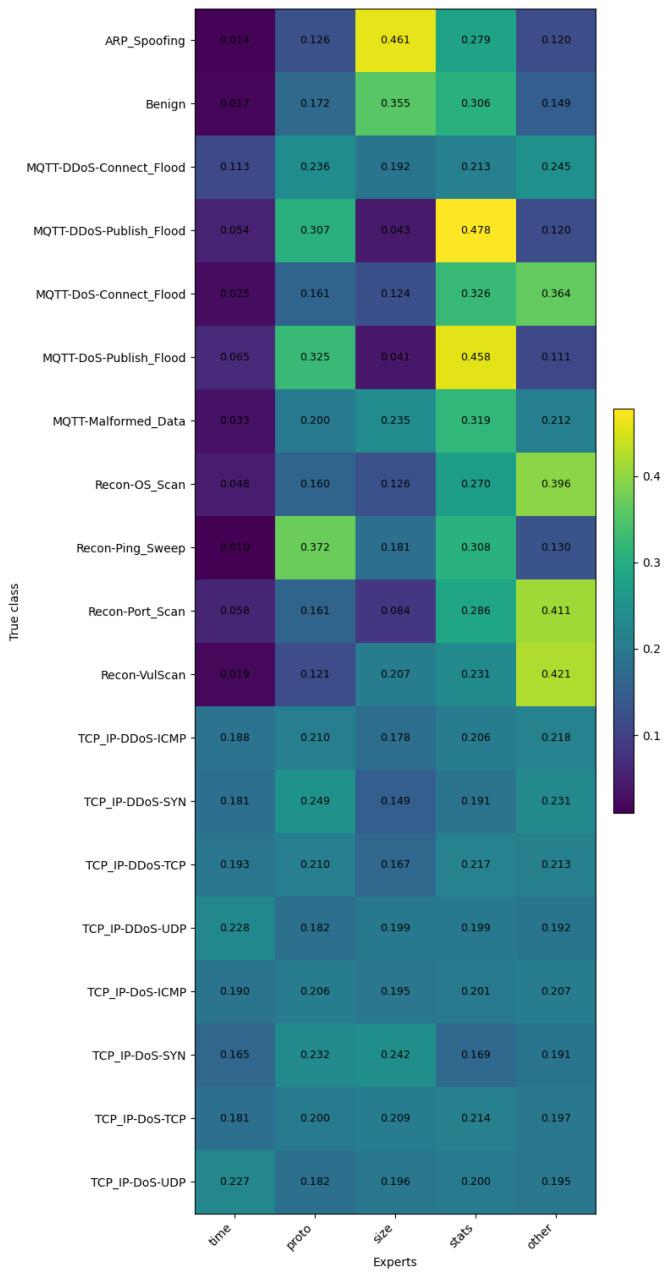
Gate heatmap illustrating expert routing behavior on the CICIoMT2024 under 19-class classification task.

**Figure 13:**
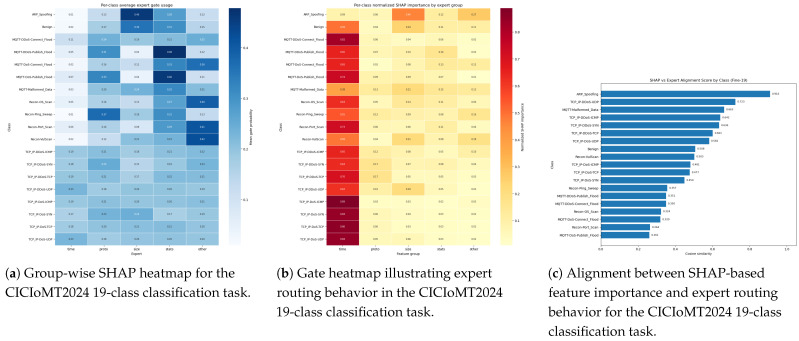
Interpretability analysis of the proposed model on the CICIoMT2024 19-class classification task.

The 19-class LIME explanations shown in Figure 11a,b highlight subtype-specific decision patterns. For *MQTT-DDoS-Connect_Flood*, the prediction is mainly driven by packet-flag and timing-related features such as *fin_flag_number*, *ack_count*, and *IAT*. In contrast, the *Recon-OS_Scan* explanation is influenced more by features such as *syn_flag_number* and *Magnitude*, while protocol-related attributes such as *SMTP*, *Telnet*, and *ARP* oppose the decision. This confirms that the model learns distinct local evidence for different fine-grained attack subtypes. Figure 12 shows that the *time* group dominates most TCP/IP attack subclasses, while *ARP_Spoofing* depends more strongly on the *size* group. Several MQTT and reconnaissance-related classes exhibit a more distributed importance profile across *proto*, *stats*, and *other*, indicating subtype-dependent feature-group relevance. Figure 13a–c provide an expert-level view of the fine-grained decision process. The per-class SHAP-group heatmap shows that the *time* group dominates most TCP/IP DoS and DDoS subclasses, whereas classes such as *ARP_Spoofing* rely more strongly on the *size* group and some MQTT or reconnaissance classes show more distributed importance across *proto*, *stats*, and *other*. The gate-usage heatmap reveals corresponding class-dependent routing behavior: *ARP_Spoofing* and *Benign* are routed more strongly toward the *size* and *stats* experts, MQTT flood variants show stronger preference for *stats*, *proto*, and *other* experts, while many TCP/IP attack subclasses exhibit a more uniform routing profile. Finally, the SHAP–expert alignment scores show that the agreement between feature-group importance and expert routing is class dependent, with the strongest alignment observed for *ARP_Spoofing* and several TCP/IP DDoS subclasses. Overall, these results indicate that the proposed MoE layer does not behave uniformly across classes, but instead provides meaningful subtype-sensitive routing behavior that complements the feature-level explanations.

### 5.3. Results on IoMT-TrafficData

#### 5.3.1. Classification Performance Analysis

**Figure 14:**
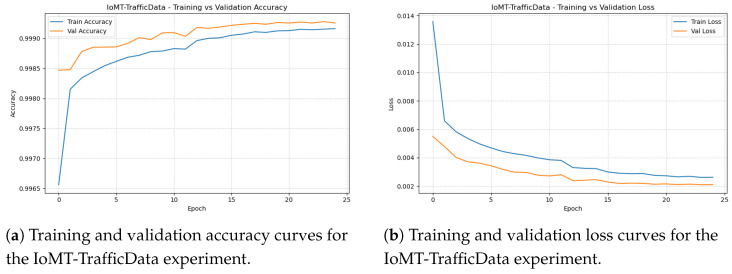
Training behavior of the proposed model on the IoMT-TrafficData dataset.

**Figure 15:**
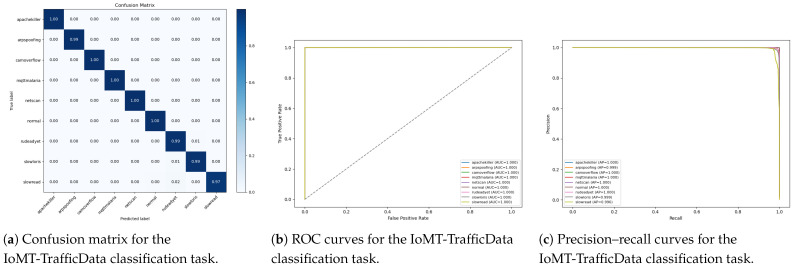
Performance evaluation of the proposed model on the IoMT-TrafficData classification task. (**a**) Confusion matrix. (**b**) ROC curves. (**c**) Precision–recall curves.

This subsection evaluates the proposed IDS framework on the IoMT-TrafficData dataset under the 9-class multiclass setting. After removing the leakage-prone helper field is_attack, the model was trained on the flow-based traffic representation defined by the traffic label. As shown in Figure 14a,b, the model converges rapidly and then remains highly stable across the remaining epochs. The training and validation accuracy curves increase smoothly toward near-saturated values, while the corresponding loss curves decrease consistently without instability. The close agreement between the training and validation trajectories indicates that the proposed architecture learns robust flow-level representations and generalizes well on this dataset. The normalized confusion matrix in Figure 15a shows that the proposed framework achieves nearly perfect discrimination for most classes. In particular, *apachekiller*, *camoverflow*, *mqttmalaria*, *netscan*, and *normal* are classified almost perfectly, while only minor confusion is observed among a few behaviorally closer classes. The most noticeable residual errors occur between *rudeadyet* and *slowloris*, and to a lesser extent between *slowread* and *rudeadyet*. These patterns indicate that the remaining errors are concentrated in classes with more similar traffic characteristics rather than in the dominant attack categories. Overall, the confusion matrix confirms that the proposed model provides very strong multiclass discrimination on IoMT-TrafficData. Furthermore, the ROC and Precision–Recall curves in Figure 15b,c further support this conclusion. The ROC curves are essentially saturated for all classes, with class-wise AUC values of approximately 1.000, indicating extremely strong separability in the one-vs-rest setting. The Precision–Recall curves provide a more informative perspective on the remaining hard cases and show that most classes retain near-ideal average precision, while *slowread*, *slowloris*, and *arpspoofing* exhibit slightly weaker but still very strong profiles. These results confirm that the proposed framework achieves highly reliable performance on the dataset, with only limited degradation on a few relatively harder classes. Table 7 compares the proposed IDS with LR, NB, SVM, and DNN on the IoMT-TrafficData dataset [44]. The proposed model achieves the best overall performance, with 99.92% accuracy and 99.42% for precision, recall, and F1-score. These results indicate that the proposed framework provides highly effective multiclass intrusion detection on IoMT-TrafficData.

Table 8 presents the per class performance of the proposed framework on the IoMT-TrafficData dataset. The proposed framework achieves an overall accuracy of 99.92% and an MCC of 0.9988, indicating excellent detection performance across all attack categories. Most classes achieve near-perfect precision, recall, and F1-scores, demonstrating the effectiveness of the proposed expert-based architecture in distinguishing diverse IoMT attacks. The high macro-averaged precision (0.9938), recall (0.9937), and F1-score (0.9937) further confirm the robustness and generalization capability of PAM-XMoE for multiclass IoMT intrusion detection.

#### 5.3.2. Explainability and Interpretability Analysis

**Figure 16:**
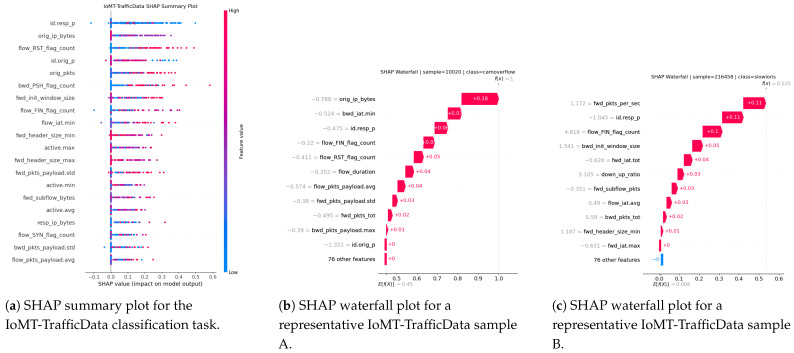
SHAP-based global and local explanations for the IoMT-TrafficData classification task. (**a**) SHAP summary plot showing global feature importance. (**b**,**c**) SHAP waterfall plots illustrating local feature contributions for two representative predictions.

The SHAP summary plot in Figure 16a shows that the model relies on a compact set of influential flow attributes, led by *id.resp_p*, *orig_ip_bytes*, *flow_RST_flag_count*, *id.orig_p*, and *orig_pkts*. This indicates that the model primarily distinguishes traffic classes through endpoint-port behavior, byte-level flow characteristics, and flag-related activity. The representative SHAP waterfall plots in Figure 16b,c provide additional local insight. In the correctly classified *camoverflow* example, the prediction is mainly driven by features such as *orig_ip_bytes*, *bwd_iat.min*, and *id.resp_p*, whereas in the *slowloris* example the decision is supported by *fwd_pkts_per_sec*, *id.resp_p*, and *flow_FIN_flag_count*. These examples show that different traffic classes are explained by distinct local feature combinations rather than by a single uniform decision pattern.

**Figure 17:**
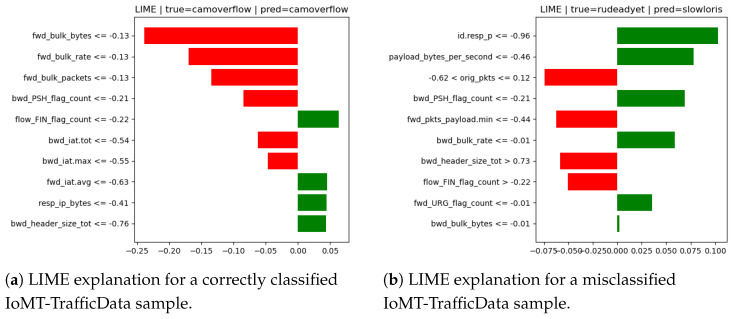
LIME-based local explanations for representative correct and incorrect predictions in the IoMT-TrafficData task.

The LIME explanations in Figure 17a,b provide complementary sample-level evidence. In the correctly classified *camoverflow* example, the decision is influenced by a combination of bulk-traffic and backward-packet features, including *fwd_bulk_bytes*, *fwd_bulk_rate*, and *flow_FIN_flag_count*. In contrast, the misclassified example, where a true *rudeadyet* sample is predicted as *slowloris*, is driven by features such as *id.resp_p*, *payload_bytes_per_second*, and *bwd_PSH_flag_count*, while other packet- and header-related attributes contribute in the opposite direction. This local explanation is useful because it clarifies why the two classes can overlap in difficult cases despite the strong overall performance.

**Figure 18:**
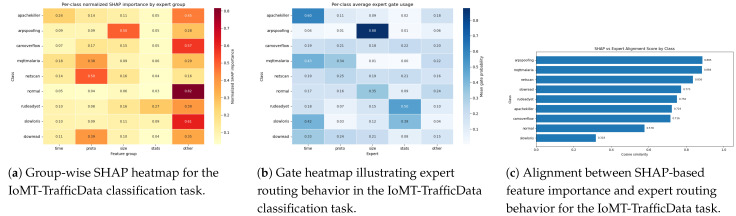
Interpretability analysis of the proposed model on the IoMT-TrafficData dataset.

Figure 18a–c provide an expert-level view of the decision process. The SHAP-group heatmap shows that different classes depend on different grouped representations: *arpspoofing* is driven mainly by the *size* group, *mqttmalaria*, *netscan*, and *slowread* rely more strongly on the *proto* group, while *apachekiller*, *camoverflow*, *normal*, and *slowloris* depend most on the *other* group. The gate-usage heatmap reveals corresponding class-dependent routing behavior: *apachekiller*, *mqttmalaria*, and *slowloris* are routed more strongly toward the *time* expert, *arpspoofing* toward the *size* expert, and *rudeadyet* toward the *stats* expert, while some classes such as *camoverflow* and *netscan* exhibit a more distributed routing profile. The SHAP–expert alignment scores further show that the agreement between feature-group importance and expert routing is strongest for *arpspoofing*, *mqttmalaria*, and *netscan*, whereas *slowloris* shows noticeably weaker alignment. Overall, these results indicate that the proposed Mixture-of-Experts layer provides meaningful class-sensitive routing behavior that complements the feature-level explanations and strengthens the interpretability of the model on IoMT-TrafficData.

### 5.4. Ablation Study

The ablation results in Table 9 demonstrate the contribution of each proposed component across the CICIoMT2024 family-level, CICIoMT2024 fine-level, and IoMT-TrafficData classification tasks. The full model consistently achieves the best overall performance, obtaining accuracies of 0.9976, 0.9907, and 0.9992 with macro F1-scores of 0.9291, 0.8428, and 0.9937 on the three datasets, respectively. These results confirm the complementary roles of the MoE gate, cross-expert attention, and the proposed classification architecture. For the CICIoMT2024 family-level classification task, removing the MoE gate, removing cross-expert attention, or replacing the attention mechanism with simple concatenation results in only marginal reductions in accuracy (0.9939–0.9955) but noticeable decreases in macro precision, recall, and F1-score compared with the full model. In particular, the macro F1-score decreases from 0.9291 to 0.8688, 0.8666, and 0.8685, respectively, indicating that the proposed gating and attention mechanisms primarily enhance balanced recognition across different traffic families rather than only improving overall accuracy. The importance of these components becomes more pronounced in the CICIoMT2024 fine-level classification task, which involves distinguishing a larger number of attack categories. While the ablated variants maintain accuracies above 0.9867, their macro F1-scores decrease to 0.7699, 0.7631, and 0.7688 compared with 0.8428 for the full model. The larger gap between accuracy and macro metrics suggests that the proposed MoE gate and cross-expert attention significantly improve the detection of minority and fine-grained attack classes, leading to a more balanced classification performance. The standard MLP classifier exhibits the weakest performance on CICIoMT2024, achieving accuracies of 0.8133 and 0.8063 with macro F1-scores of 0.7710 and 0.6253 for the family-level and fine-level settings, respectively. The substantial degradation relative to the full model demonstrates that a conventional classifier is insufficient to capture the heterogeneous and complex characteristics of IoMT traffic without the proposed expert specialization and adaptive feature interaction mechanisms. On the IoMT-TrafficData dataset, all variants achieve very high performance, with macro F1-scores ranging from 0.9882 to 0.9911, indicating that the dataset is comparatively less challenging. Nevertheless, the full model still consistently provides the highest accuracy (0.9992), macro precision (0.9938), macro recall (0.9937), and macro F1-score (0.9937), outperforming all ablated versions. Overall, the ablation study verifies that the MoE gate, cross-expert attention, and specialized expert learning jointly enhance the robustness, balanced classification capability, and generalization performance of the proposed IoMT intrusion detection framework across datasets with varying levels of classification complexity.

### 5.5. Comparison of Proposed IDS Against Representative IoMT Intrusion Detection Methods

Table 10 presents a comparative evaluation of the proposed PAM-XMoE model against representative IoMT intrusion detection methods. The results show that the proposed approach significantly outperforms existing techniques, including HCLR-IDS [20], LSTM [21], and ConvNext–MoEs [29]. In particular, while LSTM achieves competitive performance, it lacks generalization across all metrics, and both HCLR-IDS and ConvNext–MoEs show relatively lower detection capability, especially in recall and F1-score. In contrast, the proposed PAM-XMoE consistently achieves the best performance across all evaluation metrics, demonstrating its superior ability to capture complex IoMT traffic patterns and improve detection reliability.

## 6. Conclusions

This paper presented an explainable hybrid intrusion detection framework for IoMT environments. The proposed framework integrates feature-group-aware expert learning, gate-balanced MoE routing, and cross-expert self-attention to capture complementary traffic characteristics from heterogeneous IoMT network traffic. In addition to predictive modeling, the framework incorporates multi-level interpretability through SHAP, LIME, and expert-routing analysis, enabling both feature-level and internal model-behavior interpretation. The effectiveness of the proposed framework was validated on two recent IoMT benchmark datasets, namely CICIoMT2024 and IoMT-TrafficData. On CICIoMT2024, the model achieved 99.76% accuracy, 92.91% macro F1-score, and an MCC of 0.9951 in the 6-class classification setting, while attaining 99.07% accuracy, 84.29% macro F1-score, and an MCC of 0.9892 in the 19-class classification setting. On IoMT-TrafficData, the proposed framework achieved 99.92% accuracy, 99.37% macro F1-score, and an MCC of 0.9988 for the multiclass task. These results demonstrate that the proposed architecture is highly effective for both coarse-grained and fine-grained multiclass intrusion detection under heterogeneous IoMT traffic conditions. The interpretability analysis further showed that the framework does not operate as a purely black-box detector. SHAP and LIME identified meaningful traffic attributes that influence both global and local predictions, while the expert-routing analysis revealed class-dependent expert utilization and meaningful alignment between feature-group importance and MoE routing behavior. Therefore, the proposed framework provides not only strong detection capability but also improved transparency for security analysts and healthcare stakeholders. Overall, the findings indicate that the proposed Hybrid-IDS offers an effective and interpretable solution for IoMT intrusion detection. Future work will focus on extending the framework to additional IoMT datasets, improving minority-class discrimination in fine-grained settings, and investigating lightweight deployment for real-time medical network environments.

### Limitations and Future Research Directions

Despite the promising performance achieved by the proposed IDS, several limitations should be acknowledged. First, the proposed framework introduces additional computational overhead compared with conventional machine learning and single-network deep learning models due to the use of multiple expert networks, adaptive gating, and cross-expert attention mechanisms. Although the model remains relatively compact in size, training time increases with dataset scale and model complexity. Second, while focal loss and gate-balanced routing help mitigate class imbalance, performance degradation can still occur for minority classes and highly similar attack categories, particularly in fine-grained multiclass settings. This challenge remains an open issue for IoMT intrusion detection and warrants further investigation. Third, the scalability of the proposed expert-based architecture to substantially larger feature spaces, larger numbers of traffic classes, or continuously evolving attack types has not yet been fully explored. Additional studies are required to assess the behavior of the routing mechanism and expert specialization under more complex deployment scenarios. Finally, although the proposed framework demonstrates strong performance on benchmark datasets, real-world IoMT environments may introduce practical challenges related to resource constraints, latency requirements, dynamic traffic distributions, and previously unseen attacks. Future work will therefore focus on lightweight model optimization, real-time edge deployment, continual learning strategies, and evaluation on additional real-world IoMT datasets.

## Figures and Tables

**Table 1:** Summary of recent DL-based intrusion detection approaches for IoMT/IoT environments.

Ref.	Model	Key Features	Limitations
[20]	HCLR-IDS	Combines CNN, LSTM, DQN, and PPO for IoMT intrusion detection; applies Enhanced Mutual Information Feature Selection (MIFS) on CICIoMT2024, captures both spatial and temporal traffic patterns.	Multiclass performance remains relatively lower for fine-grained attack detection; reinforcement learning components increase model complexity.
[21]	Blockchain + MA-DeepCRNN IDS	Integrates blockchain with a multi-attention CNN-RNN framework for secure IoMT intrusion detection, evaluated on CICIoMT2024 with strong binary, 6-class, and 19-class results.	Blockchain integration may increase computational overhead and deployment complexity, especially in resource-constrained environments.
[22]	Enhanced LSTM-based IDS	Employs a simpler LSTM-based architecture for IoMT attack classification on CICIoMT2024, achieving strong 19-class classification performance.	Relies mainly on sequential modeling and may have limited ability to capture complex global feature interactions.
[23]	Hybrid CNN-LSTM	Combines CNN for local feature extraction and LSTM for temporal dependency modeling, evaluated on IoT-23, N-BaIoT, and CICIDS2017.	Focuses on general IoT rather than IoMT-specific traffic, lacks interpretability, and protocol-aware modeling.
[24]	MF-Transformer	Introduces Memory Feedback LSTM throughout Transformer layers to capture spatial and temporal dependencies, evaluated on WUSTL-EHMS-2020, ECU-IoHT, and X-IIoTID.	Transformer-based design may still involve relatively high computational cost; evaluation is not centered on CICIoMT2024-like protocol-diverse IoMT traffic.
[25]	Lightweight Transformer-based IDS	Combines depth-wise separable convolutions with a two-layer Transformer encoder; uses packet and flow-level features, includes explainability analysis.	Although lightweight, it may still have limited specialization for highly heterogeneous IoMT protocols and attack behaviors.
[26]	Transformer + SHAP for Spoofing Detection	Uses a Transformer-based framework with custom attention and SHAP explainability; addresses class imbalance with SMOTE-Tomek, focuses on spoofing attack detection in CICIoMT2024.	Specialized for spoofing attack detection only, less suitable for general multiclass IoMT intrusion detection.
[27]	TabTransformer + Random Forest	Hybrid framework for biometric healthcare security, combines TabTransformer and Random Forest; integrates SHAP, LIME, ELI5, and permutation importance for interpretability.	Primarily focuses on DDoS/DoS detection and biometric-data security; narrower attack coverage than general IoMT IDS frameworks.
[28]	MedMixtral 8x7B	MoE-based medical large language model with an offloading strategy for IoMT-enabled e-healthcare; emphasizes privacy protection and efficient deployment.	Not primarily designed for intrusion detection, focuses on medical question answering and deployment efficiency rather than IDS tasks.
[29]	ConvNeXt + Sparse BiLSTM Experts + Symmetric Linear Routing	Combines ConvNeXt, sparse Mixture of Experts, and Top-K gated BiLSTM experts for IoT threat detection; evaluated on CIC-IDS2018, TON-IoT, and BoT-IoT.	Designed for broader IoT settings rather than specifically for IoMT, with a limited focus on interpretability.
[30]	CPS-oriented IDS with MoE and CPS-SNORT	Integrates signature-based detection, MoE, CPS-SNORT rules, and LSTM-based anomaly detection for CPS protection, targeting unsafe state prevention.	Host-based and CPS-specific; less directly applicable to healthcare-oriented IoMT network intrusion detection.
[31]	RF/XGBoost + TCN for Ransomware Detection	Combines Random Forest or XGBoost with TCN to model the temporal progression of ransomware risk signals in IoMT environments.	Evaluated on a synthetic IoMT ransomware dataset, limited scope compared with broader multiclass intrusion detection frameworks.

**Table 2:** Instance distribution of the datasets used in this study.

Dataset	Experimental Setting	Class	Instances		
			Train	Test	Total
CICIoMT2024	6-class	Benign	192,732	37,607	230,339
		Spoofing	16,047	1744	17,791
		Recon	103,726	27,676	131,402
		MQTT	262,938	63,715	326,653
		DoS	1,805,529	416,676	2,222,205
		DDoS	4,779,859	1,066,764	5,846,623
		Total	7,160,831	1,614,182	8,775,013
IoMT-TrafficData	Multiclass	camoverflow	–	–	1,640,039
		normal	–	–	766,915
		netscan	–	–	467,093
		rudeadyet	–	–	131,081
		apachekiller	–	–	84,579
		mqttmalaria	–	–	69,623
		slowloris	–	–	63,608
		arpspoofing	–	–	11,236
		slowread	–	–	9014
		Total	–	–	3,243,188

**Table 3:** Main hyperparameter settings used in the experiments.

Hyperparameter	Value
Optimizer	Adam
Learning rate	$3\times 10^{-4}$
Batch size	64
Dropout rate	0.25
Training epochs	25
Validation ratio	0.15
Test ratio (IoMT-TrafficData)	0.20
Gate temperature	1.05
Gate balance coefficient	0.002
Gate entropy coefficient	0.0005

**Table 4:** Comparison of baseline models and the proposed IDS on the CICIoMT2024 dataset under the 6-class and 19-class classification setting.

Dataset Setting	Model	Accuracy	Precision	Recall	F1-Score
CICIoMT2024 6-class	LR	0.743995	0.732297	0.609011	0.604438
	AB	0.903036	0.841781	0.775371	0.805621
	DNN	0.780524	0.768093	0.760205	0.733549
	Proposed IDS	0.997600	0.938100	0.921500	0.929100
CICIoMT2024 19-class	LR	0.745625	0.578141	0.745625	0.430982
	AB	0.236537	0.501689	0.341710	0.301097
	DNN	0.776566	0.738350	0.601949	0.579062
	Proposed IDS	0.990700	0.911200	0.832900	0.842800

**Table 5:** Per class Performance on the CICIoMT2024 dataset under the 6-class classification setting.

Metric	Benign	DDoS	DoS	MQTT	Recon	Spoofing
Precision	0.9451	0.9995	0.9999	0.9982	0.9763	0.7098
Recall	0.9851	0.9999	0.9988	0.9914	0.9442	0.6101
F1-score	0.9647	0.9997	0.9993	0.9948	0.9600	0.6562
Overall Performance						
Accuracy	0.9976					
Precision (Macro)	0.9381					
Recall (Macro)	0.9215					
F1-Score (Macro)	0.9291					
MCC	0.9951					
Model Size (MB)	0.267864					
Inference Time (ms/sample)	0.049					

**Table 6:** Per-class performance on CICIoMT2024 under the 19-class classification setting.

Class	Precision	Recall	F1-Score
ARP-Spoofing	0.7758	0.6508	0.7078
Benign	0.9414	0.9934	0.9667
MQTT-DDoS-Connect Flood	0.9989	0.9999	0.9994
MQTT-DDoS-Publish Flood	0.9969	0.1143	0.2051
MQTT-DoS-Connect Flood	0.9997	0.9844	0.9920
MQTT-DoS-Publish Flood	0.5330	0.9999	0.6953
MQTT-Malformed Data	0.8979	0.6846	0.7769
Recon-OS Scan	0.8051	0.3727	0.5095
Recon-Ping Sweep	0.9533	0.7688	0.8512
Recon-Port Scan	0.9154	0.9611	0.9377
Recon-VulScan	0.5055	0.3095	0.3839
TCP/IP-DDoS-ICMP	0.9991	0.9997	0.9994
TCP/IP-DDoS-SYN	0.9995	0.9930	0.9962
TCP/IP-DDoS-TCP	0.9942	0.9994	0.9968
TCP/IP-DDoS-UDP	0.9985	0.9994	0.9990
TCP/IP-DoS-ICMP	0.9997	0.9984	0.9990
TCP/IP-DoS-SYN	0.9999	0.9992	0.9995
TCP/IP-DoS-TCP	0.9999	0.9992	0.9996
TCP/IP-DoS-UDP	0.9996	0.9987	0.9991
Recall (Macro)	0.8329		
F1-Score (Macro)	0.8428		
MCC	0.9891		
Model Size (MB)	0.27108		
Inference Time (ms/sample)	0.052		

**Table 7:** Performance comparison of baseline models and the proposed IDS on the IoMT-TrafficData dataset.

Model	Accuracy	Precision	Recall	F1-Score
LR	96.95	97.13	96.95	97.04
NB	92.50	92.97	92.50	92.61
SVM	96.93	96.99	96.93	96.96
DNN	97.63	97.80	97.63	97.71
Proposed IDS	99.92	99.38	99.37	99.37

**Table 8:** Per-class performance of the proposed framework on the IoMT-TrafficData dataset.

Metric	Apachekiller	Arpspoofing	Camoverflow	Mqttmalaria	Netscan	Normal	Rudeadyet	Slowloris	Slowread
Precision	0.9992	0.9850	1.0000	0.9996	0.9999	0.9999	0.9921	0.9844	0.9837
Recall	0.9989	0.9947	1.0000	0.9997	0.9997	1.0000	0.9915	0.9866	0.9723
F1-score	0.9991	0.9898	1.0000	0.9997	0.9998	0.9999	0.9918	0.9855	0.9780
Accuracy	0.9992								
Precision (Macro)	0.9938								
Recall (Macro)	0.9937								
F1-Score (Macro)	0.9937								
MCC	0.9988								
Model Size (MB)	0.28911								
Inference Time (ms/sample)	0.004								

**Table 9:** Ablation study results on CICIoMT2024 family-level and fine-level classification settings.

Dataset Setting	Variant	Accuracy	Prec.	Rec.	F1
CICIoMT2024 6-class	*w*/*o* MoE gate	0.995424	0.858462	0.907075	0.868757
	*w*/*o* cross-expert attention	0.993948	0.856890	0.913159	0.866556
	simple concatenation fusion	0.995502	0.857353	0.905846	0.868540
	standard MLP classifier	0.813347	0.795460	0.803982	0.770950
	Full Model	0.997600	0.938100	0.921500	0.929100
CICIoMT2024 19-class	*w*/*o* MoE gate	0.987166	0.874657	0.786026	0.769879
	*w*/*o* cross-expert attention	0.986738	0.899108	0.779163	0.763127
	simple concatenation fusion	0.987506	0.874869	0.780899	0.768782
	standard MLP classifier	0.806259	0.765444	0.647818	0.625252
	Full Model	0.990700	0.911200	0.832900	0.842800
IoMT-Traffic Data 9-class	*w*/*o* MoE gate	0.998770	0.990432	0.988142	0.989201
	*w*/*o* cross-expert attention	0.998731	0.991652	0.988561	0.990019
	simple concatenation fusion	0.998916	0.991800	0.990433	0.991070
	standard MLP classifier	0.998468	0.989687	0.986935	0.988200
	Full Model	0.999200	0.993800	0.993700	0.993700

**Table 10:** Comparison with representative IoMT intrusion detection methods with proposed IDS.

Reference	Model	Accuracy	Precision	Recall	F1-Score
[20]	HCLR-IDS	0.7773	0.7386	0.6016	0.5829
[21]	LSTM	0.9800	0.9800	0.9800	0.9800
[29]	ConvNext–MoEs	0.9408	0.9368	0.9408	0.9322
Proposed IDS	PAM-XMoE	0.9992	0.9938	0.9937	0.9937

## Data Availability

The link to the dataset used in this study is provided in the paper.
